# Erectile Dysfunction in Pelvic Cancer Survivors and Current Management Options

**DOI:** 10.3390/jcm12072697

**Published:** 2023-04-04

**Authors:** Jose Bernal, Krishnan Venkatesan, Francisco E. Martins

**Affiliations:** 1 Department of Urology, Hospital Sotero del Rio/Clinica Indisa, Santiago 13123, Chile; 2 Department of Urology, MedStar Washington Hospital Center, Washington, DC 20010, USA; 3 Department of Urology, School of Medicine, University of Lisbon, Hospital Santa Maria, 1649-035 Lisbon, Portugal; faemartins@gmail.com

**Keywords:** erectile dysfunction, pelvic surgery, radical pelvic surgery, penile rehabilitation, prostate cancer, bladder cancer, rectal cancer

## Abstract

Pelvic malignancies, including prostate, rectal, and bladder cancers, are among the most frequent malignancies found in the male population. These issues are most effectively and commonly treated with radiotherapy and/or surgery. However, these treatments can cause collateral damage, resulting in significant impacts on quality of life, with erectile dysfunction being one of the most frequent postoperative complications. Currently, there are several treatment options for erectile dysfunction, including oral phosphodiesterase type 5 inhibitors, vacuum erection devices, intracorporeal injections, and penile prosthesis. The latter has shown to be an effective and safe technique, with results comparable to those obtained by patients without pelvic surgery or radiotherapy. The results of early penile rehabilitation programs are promising and they have been incorporated into a greater proportion of treatment plans more recently, with varying degrees of success. In this narrative review, we summarize the literature on erectile dysfunction after pelvic cancer treatments and its management.

## 1. Introduction

Erectile dysfunction (ED) is defined as the inability to attain or maintain a penile erectile (EF) function sufficient to permit sexual relations [1].

There are many risk factors associated with ED including diabetes, smoking, and hypertension. These conditions are known to cause damage to vasculature and nerves, resulting in vascular and neurogenic ED [2].

Anatomical descriptions of pelvic nerves have helped to shape our understanding of the impact of pelvic surgery on sexual function. Damage—whether transection, thermal, ischemic, or traction related—to parasympathetic nerves along their path from the spinal cord to the penis will affect erectile function, while damage to sympathetic nerves will affect ejaculation [3,4].

The cavernous nerve is the primary autonomic nerve involved in erection it and originates from the sympathetic pelvic plexus (arising from T11–L2 level) and the parasympathetic nerves (arising from S2–S4 level and as the inferior hypogastric plexus lies on the rectum in men) [5,6]. As a process, the cavernous nerve travels between the prostatic fascia and levator fascia and meets with branches of the hypogastric vasculature to form the neurovascular bundle (NVB), which enters the corpora cavernosum via the tip of the prostate, carrying and releasing the neurotransmitters responsible for erection [3].

In response to different stimuli, the parasympathetic nerves release acetylcholine. This stimulates the release of nitric oxide at the level of endothelial cells in corpora cavernosa. Nitric oxide activates the guanylate cyclase that increases cGMP. This decreases intracellular calcium concentrations, leading to smooth muscle relaxation. Consequently, blood flow within the sinusoids increases significantly and makes the penis rigid. In the corpora cavernosa, which are surrounded by the tunica albuginea, pressure increases and emissary veins are compressed, limiting outflow and resulting in the maintenance of tumescence. The phosphodiesterase enzymes counteract the effect of guanylate cyclase by hydrolyzing cGMP, causing detumescence of the penis [5,7].

After pelvic surgery, nocturnal erections are absent due to temporary or permanent injury to the cavernous nerve [3,4]. The absence of erections decreases cavernous oxygenation, promoting smooth muscle apoptosis, collagen deposition, and fibrosis. These fibrotic changes disable compression of the emissary veins running under Buck’s fascia, leading to veno-occlusive dysfunction and (venous leak) ED [3,4,8,9].

Penile denervation in rats has been demonstrated to initiate smooth muscle apoptosis in the cavernous tissue, an outcome which is likely due to the loss of growth factors produced by the cavernous nerve [4,10].

Vascular dysfunction can also be the result of cavernous artery insufficiency [4]. Erectile tissue requires oxygenation to maintain its integrity and interruption of arterial flow, particularly disruption to the accessory pudendal arteries (present in 4–75% of men) [11] that run parallel to the NVB, can affect potency. Mulhall et al. reported that 59% of patients have developed arterial insufficiency after radical prostatectomy, while they also report that arterial preservation can shorten the recovery time and improve erectile function [4,12]. However, other studies on robotic-assisted radical prostatectomy have indicated that transection of an accessory pudendal artery has no impact on the recovery of the capacity to have erections or of potency [11,13,14].

### 1.1. Pelvic Surgeries Associated with Erectile Dysfunction

Pelvic surgeries are among the most common causes of ED in men [15]. The pathophysiology of ED is multifactorial, with vascular, neurogenic, and psychological components [4,6,15]. The pelvic surgeries most often associated with ED include radical prostatectomy (RP), radical cystectomy (RC), and low anterior resection (LAR or abdominoperineal resection (APR) for rectal cancer [2,15].

### 1.2. Radical Prostatectomy

Prostate cancer (PCa) is the second most frequently observed neoplasia (after lung cancer) in males worldwide [16,17,18,19].

As screening for prostate cancer has increased in the past few decades, prostate cancer has generally been detected at an earlier stage and in younger patients. As treatment has also been refined, with more patients surviving longer, quality of life concerns have become central [2,8,19].

Radical prostatectomy remains the most common treatment option for localized PC [2,17,20,21]. However, the treatment carries with it significant sexual side effects [2].

With continuously improving knowledge of pelvic anatomy, progressive changes have been implemented in the RP surgical technique in order to reduce complications, such as ED. One of the most important was described by Walsh and Donker in 1982 in their description of the nerve-sparing (NS) technique. Namely, it involves making an anterolateral incision in the pelvic fascia, parallel to the neurovascular bundles, to avoid their injury [2,22,23].

Currently, there are three available options for the surgical extirpation of the prostate: robot-assisted RP (RALP), laparoscopic RP (LRP), and traditional open RP (ORP) [17]. The reported rates of erectile dysfunction after bilateral nerve-sparing, open, or minimally invasive RP, vary in most series between 24 and 66% [2,11,20,24,25].

The wide variability in reported ED rates is attributed to many factors including surgical technique (with or without nerve-sparing procedures), single- vs. multi-surgeon series, surgeon volume or experience, definition of ED, length of follow-up, variable patient demographics, differences in data acquisition technique (different questionnaires used and the definition of quality of erection), amongst others [11,26]. It is crucial to appropriately estimate the prevalence of ED in order to improve medical decision making, counsel patients, and set realistic expectations [26].

In the past decades, there have been many advances with the introduction of new technologies resulting in the development of less invasive and more precise surgical techniques. LRP and RALP were first introduced in 1999 and 2000, respectively [4]. Robotic surgery provides an improved 10-times-magnified three-dimensional vision and the instruments enhance dexterity through seven degrees of motion, assisting in the development of a more anatomical dissection that can limit impact or injury to the neurovascular bundles [4,25].

RALP has been widely adopted and popularized worldwide and today is the most common surgical approach [17,27,28]. However, comparisons of the outcomes of the 3 techniques in different retrospective, prospective, controlled studies or meta-analyses are inconclusive and most have not shown any specific technique to have a statistically significant advantage (survival or otherwise) [4,17,25,28,29,30,31,32,33].

Coughlin et al. presented the first randomized controlled trial, including 296 men, to compare the postoperative functional results of RALP with open radical retropubic prostatectomy at 6, 12 and 24 months. Sexual function scores were not significantly different between RALP vs. ORP (EPIC score: 45.7 vs. 46.9; IIEF: 33.9 vs. 33.8) [28].

In their study including 10,790 patients, Haese et al. showed that recovery of erectile function was similar between RALP and ORP at 12 months (86.3% vs. 80.3%, respectively) [31]. In their meta-analysis of the outcomes of minimally invasive techniques (RALP or LRP) compared with ORP, Lan Cao et al. showed that the potency recovery rates at 12 months postoperatively were 14.6% and 20.3%, with no significant difference, respectively [30].

Other perspectives and meta-analysis studies favored RALP over LRP and ORP [8,27,30,34,35]. In a meta-analysis including 227,400, patients Basiri et al. showed that the rate of ED was significantly lower after RALP than after LRP; however, it was not significantly different between ORP and RALP [36].

Haglind et al. (LAPPRO group) carried out a large multicenter prospective, controlled, nonrandomized trial, including 2431 men, to compare ED at 12 months after RALP or ORP treatment. Using an IIEF questionnaire, ED was found to be significantly less frequent in RALP group (70.4% vs. 74.7%; OR 0.81) [37]. This difference was maintained (66% vs. 70%) at 8 years of follow-up [27].

Studies have identified different factors (pre-surgical, intra-surgical, and post-surgical) that influence the recovery of sexual potency after NS surgery. The most important factors are preoperative erectile function and age [8,11,35,38,39].

As with PCa incidence, the prevalence of ED independently increases with age. Specifically, 20–40% of men between the ages of 60 and 69 years old have ED and between 50–100% of men in their 70s and 80s have the condition [40]. Men with preoperative ED cannot expect an improvement in their baseline erectile function [4]. Other factors affecting ED are comorbid diseases (diabetes, hypertension, cardiovascular diseases), lower prostate-specific antigen levels, lower cancer stage, pretreatment IPSS, nerve-sparing surgical techniques, the surgeon’s experience, higher preoperative serum testosterone, and prostate size [2,4,8,20,24,35,38,41,42,43]. Post-surgical factors such as the presence of urinary incontinence and the need for adjuvant treatments (radiation and hormonal therapy) also portend a higher risk of ED [2].

Nomograms have been developed using these factors to predict the recovery of erectile function after radical prostatectomy to aid in thorough patient counseling [44,45,46].

In a recent investigation including 17,250 patients with prostate cancer, Pellegrino et al. were the first to assess the prevalence of ED (using IIEF) in specific combinations of ages and using different number of comorbidities (rather than age intervals or average comorbidity). The authors found that the risk of developing ED increased substantially with comorbidities. For example, the probability of ED occurring for 50- and 75-year old individuals was 20% and 68% for healthy men, but 41% and 85% for those with hypertension, obesity, and diabetes. All risk factors assessed, with the exception of dyslipidemia (OR 0.97), were significantly associated with ED. Diabetes was the strongest risk factor (OR 2.01), followed by anxiety/depression (OR 1.48), hypertension (OR 1.32) and cardiovascular disease (OR 1.22), among others [47].

Schmid et al. suggested that preoperative MRI of the prostate could also be used for the prediction of EF recovery after RP [48].

Here, it is important to note that the restoration of erectile function after RP does not necessarily ensure the restoration of presurgical sexual satisfaction [21].

### 1.3. Radical Cystectomy

Radical cystectomy (RC) is the gold standard management technique for treating muscle-invasive and refractory non-muscle-invasive bladder cancer [15,49,50].

Robotic-assisted laparoscopic radical cystectomy (RARC) was introduced in 2003. Although its use has rapidly increased, randomized controlled trials have shown similar perioperative outcomes and complication rates between traditional open radical cystectomy (ORC) and RARC methods [51].

In a recent multicentric randomized clinical study including 317 patients with nonmetastatic bladder cancer, Catto et al. compared morbidity after robot-assisted radical cystectomy with intracorporeal reconstruction vs. that performed with open radical cystectomy. The results showed a statistically significant increase in days alive and out of the hospital over 90 days (82 vs. 80 days, respectively). Additionally, patients who received robotic surgery had fewer thromboembolic complications (1.9% vs. 8.3%); wound complications (5.6% vs. 16.0%); and a significantly higher health-related quality of life than those patients who had undergone open surgery. The median length of stay in the hospital was 7 days for robotic radical cystectomy and 8 days for open surgery, and there were no statistically significant differences in cancer recurrence and overall mortality [52].

One of the most frequent consequences of RC is ED and it shares similar pathophysiology to ED after other extirpative cancer-related pelvic surgeries [6]. Cisplatin—commonly used in neoadjuvant or other settings for bladder cancer tratment—can also contribute to ED. Indeed, it has known testicular toxicity with resultant testosterone deficiency, a known risk factor for ED [6].

The potency rates after RC range between 14% and 80% and are comparable between ORC and RARC series [19,49,51]. Nerve-sparing techniques preserve the autonomic nerves involved in erectile function, improving potency compared to standard RC [6,50,51] without compromising oncological outcomes. Rates of post-RC ED are estimated to be 10–30% after NS techniques and 80–90% following the use of standard technique [6].

The different NS RC surgical techniques described are prostate-sparing RC, capsule-sparing RC, seminal-sparing RC, and nerve-sparing RC where only the NVB is conserved [6]. The described potency rates in the review of Pederzoli et al., are 89.7% for prostate-sparing RC, 59–93.8% for capsule-sparing RC, and 77–78.8% for nerve-sparing RC. Any nerve-preserving technique results in better potency compared to the use of standard RC [6]. Nevertheless, because of study limitations, no definitive conclusions could be drawn about the superiority of any of the NS techniques.

No studies exist which show a significant difference in ED between different types of urinary diversion [6,50]. The recovery of potency is probably more dependent on the use of NS surgery, age, and preoperative potency status than on the diversion technique itself [50].

### 1.4. Radiotherapy Treatment in Prostate Cancer

Radiotherapy (RT) is another effective treatment option in prostate cancer and is chosen by 33% and 50% of patients [19,53,54]. Ionizing radiation is employed to induce apoptosis of tumor cells [19].

Broadly speaking, RT includes external beam radiotherapy (EBRT) and brachytherapy (BT). EBRT has evolved into newer modalities such as intensity-modulated radiotherapy (IMRT), stereotactic radiotherapy (SBRT), and three-dimensional conformal radiotherapy (3DCRT). These involve a higher energy being delivered in a tighter focus of tumor tissue, with less energy affecting adjacent normal tissues (penis, bulbar urethra, NVB). This is of course designed to minimize complications or side effects. In BT, radioactive ‘seeds’ are implanted (temporarily or permanently) into the prostate tissue [19,54].

One of the most common consequences of RT is radiation-induced erectile dysfunction (RIED) [19,53]. A wide range of RIED has been reported in the literature. This can likely be explained by the multifactorial etiology of ED and the use of varied definitions of ED [53]. Nukala et al. described RIED rates between 17% and 90% [53] and a meta-analysis including 26,269 men suggested a prevalence of RIED of 34% of men at 1 year and 57% at 5.5 years [55].

Alemozaffar et al. [41] evaluated the ED rate at 24 months after treatment in a prospective, longitudinal, multicenter cohort including 1027 patients with localized prostate cancer who elected for prostatectomy, external beam radiotherapy, or brachytherapy. ED was found in 65%, 63% and in 57% of patients, respectively. Pretreatment sexual HRQOL score, age, serum prostate-specific antigen level, race/ethnicity, body mass index (BMI), and intended treatment details were all associated with functional erections 2 years after treatment. This study also developed models with which to predict long-term EF following prostate cancer treatment based on individual patient and treatment choice.

Donovan et al. evaluated outcomes using questionnaires among 1643 men: at 6 years, ED was found in 83% of the prostatectomy group, in 73% of the radiotherapy group and in 70% of the active-monitoring group [56].

A recent systematic review, including 2714 patients, discovered a direct correlation between the incidence of ED and radiation dose and length of follow-up [54]. The authors observed an increased risk of ED by 2.2% for each 1 Gy increase in dose and by 1.5% for each 1-month increase in the follow-up period [54,55]. With a median follow-up of 25 months, 57% of previously potent men conserved a normal potency. As such, RIED reporting was 17%, 26%, 23%, and 23% for 3DCRT, IMRT, low dose rate BT, and SBRT treatments, respectively [54]. There was no significant difference between radiation types/modalities regarding sexual potency [19,54,55]. The researchers concluded that the advancements in RT technologies have successfully diminished the risk of ED.

The deleterious effects of radiation on EF are delayed, unlike the immediate effects of surgery, and patients may experience a progressive deterioration of erectile function [57,58].

Potosky et al. evaluated 1187 patients at 5 years after diagnosis (RP in 901 patients vs. EBRT in 286 patients). ED was more prevalent in the RP group than in the EBRT group (79.3% versus 63.5%) and the difference was much more evident at the 2 first years after diagnosis (ED 82.1% in RP group vs. 50.3% in EBRT group) [59].

### 1.5. Colorectal Surgery

Colorectal cancer is the third most common cancer worldwide [60,61,62]. Advances in early diagnoses, surgical techniques, and oncological treatments have significantly improved patient survival rates [57,61,62]. ED is also common after colorectal surgery and is considered to be multifactorial, secondary to surgery, radiotherapy, presence of a stoma, depression, and anxiety [60,62,63,64].

The onset of ED after rectal cancer surgeries is attributed to damage to the pelvic plexus [2]. In general, the rate ED is significantly higher in rectal cancer survivors than in survivors of non-rectal colon cancer. Damage to the pelvic autonomic nerves during rectal surgery may be the main reason for this difference [61,63,64,65,66].

The rate ED after rectal cancer treatment is reported to range from 20 to 92% [63,64,66]. In two large population-based studies, rectal cancer survivors had higher rates of ED than either colon cancer survivors or the normative population. One of these demonstrated that rectal cancer survivors had a statistically significant difference in ED versus colon cancer survivors (54% vs. 25%), with a rate of 27% in the general population. This study also concluded that a history of RT and the presence of a stoma are significant independent risk factors for ED [61,62].

The type of surgical colorectal resection used influences the risk of ED. In general, there are higher rates of ED with more distal or lower resections [64]. In rectal cancer, the total mesorectal excision (TME) technique allows for greater preservation of the neurovascular supply and is associated with lower rates of ED. This is now considered the gold standard of treatment for use in ED [2,64,67].

The rate of ED after TME is estimated to be 11–19%. More extensive surgeries have higher rates of ED. These include methods such as APR, which includes a colostomy and total resection of the rectum and anus.

Combining surgery with radiation therapy carries an even higher risk of ED than surgery alone [2,64,66]. When further combined with neoadjuvant chemoradiotherapy, this resulted in greater rates of ED than neoadjuvant chemotherapy alone. Chemotherapy alone does not appear to increase the risk of ED. Systematic reviews have concluded that there is no significant difference in ED rates between laparoscopic and open surgery groups [66].

### 1.6. Treatment and Penile Rehabilitation

Despite technological and surgical techniques advances, ED is still a significant adverse effect of pelvic surgeries. Post-surgical ED is associated with vascular changes and neuropraxia that create a hypoxic environment that induces corporal fibrosis through collagen deposition in cavernosal smooth muscle cells. Thus, strategies to maintain appropriate oxygen levels in the corpora cavernosa have been suggested to aid in the prevention of corporal fibrosis and ED [9,68].

Penile rehabilitation programs are designed to counteract these deleterious effects and to stimulate the recovery of EF. Most of the evidence for penile rehabilitation has come from post-prostatectomy patients [9,39,69].

Penile rehabilitation programs comprise one or a combination of interventions, including pharmacotherapies (e.g., oral, injectable, intraurethral), devices (vacuum erection devices), or activities (e.g., pelvic floor muscle training (PFMT) and aerobic exercise) with different schedules, dosages, frequencies, and timing. No specific protocol is widely accepted [11,69].

The most commonly used methods used include the application of oral phosphodiesterase type 5 inhibitors (PDE5- I), vacuum erection devices (VEDs), intracorporeal injection (ICI) therapy, and penile prosthesis as a last resort [9].

It is important to discuss the real incidence of permanent or temporary post-RP ED with candidates for RP and highlight that, currently, there are no conclusive data to support a “better” specific surgical technique (ORP, LRP, RALP) for use to preserve EF [9].

It is recommended to evaluate basal and evolutive post-operative EF through validated questionnaires, such as with the international index of erectile function (IIEF) survey [2].

Philippou et al. highlight that analyzing efficacy of penile rehabilitation begins with defining objectively ED and the restoration of potency following pelvic surgery. Nevertheless, there is a wide variability in the literature. The Cochrane review used the IIEF-5 and IIEF questionnaires, defining males as ‘sufficient for intercourse’ when experiencing mild (IIEF-5 > 17) or no (IIEF > 19) ED and the return to sexual function as the return to baseline IIEF-5/IIEF scores [26].

### 1.7. Timing to Initiate Penile Rehabilitation

It is recommended that surgeons start the penile rehabilitation protocol as early as possible as this may result in superior long-term recovery outcomes due to the prevention or limitation of hypoxic-related irreversible structural changes in the erectile tissue [9,32].

Some authors have proposed that a theoretical benefit of the improvement of oxygenation prior to surgery would be that it makes the erectile tissue more tolerant after surgical injury. A recent double-blind, prospective, randomized study performed using tadalafil 5 mg/day was started in one group 2 weeks before surgery, and in the other group 4 weeks after NS-RALP, for a total period of 24 weeks. At 12 months, the recovery of unassisted EF was achieved in 80% and 71% of patients, respectively, with no statistically significant difference observed. Moreover, the authors concluded that preoperative tadalafil 5 mg/day may offer benefits in penile rehabilitation [70]. A systematic review informed researchers that treatments started before surgery (PFMT, oral medications, and vacuum devices) may aid in EF recovery and that frequent information delivery between the patient and partner also impact on sexual recovery [11,70]. However the recommended time to start penile rehabilitation is 1–4 weeks after RP [71].

A recent review recommended the combination of PDE5-I and VED as first-line options and the use of ICI with VED as the second-line option and that both be started one month after the surgery with a frequency of at least 3 times a week for patients with normal preoperative EF [9]. If penile rehabilitation is ineffective, or if there exists a history of severe preoperative ED, the use of a penile implant should be the next option [9]

Mulhall et al. found that patients who started interventions within 6 months (versus > 6 months) post-RP had significantly higher IIEF-5 scores (22 vs. 16, *p* < 0.001). Additionally, they underwent an increase in the rate of unassisted erections and PDE5-I-assisted erections [9,68].

### 1.8. Phosphodiesterase Type 5 Inhibitors (PDE5-I)

PDE5-I drugs inhibit PDE5 in endothelial cells of the corpus cavernosum, enhancing the effects of nitric oxide (NO). This induces an increase in cGMP that causes smooth muscle relaxation and consequently increased blood flow to the penis, resulting in and maintaining an erection [9]. However, nerve activation is required to initiate cGMP synthesis, which can explain why only 0 to 15% of the patients treated by non-NS RP respond to PDE5-I vs. 35 to 75% of those treated by NS RP [4,11,70].

PDE5-Is are considered to constitute the first-line treatment for postoperative NS RP ED due to their safety, efficacy profile and good tolerability without serious adverse events [70,72,73,74]. The use of DE5-I at any dosage and formulation is better than the use of placebo [70,72,73,75].

Usually, PDE5-Is are not very effective in the early postoperative period, with only 12% of patients responding. This is most likely because of post-surgical cavernous neuropraxia, a condition that suppresses erectile capacity and can take 12–24 months to recover [9,15].

A recent meta-analysis of 2822 patients found significant improvements in IIEF score and erectile function recovery after NS RP after daily and on-demand use of PDE5-I [74]. A recent systematic review included 2711 patients who were compared in categories of placebo, only pelvic floor muscle training and 100 mg sildenafil at regular doses (once daily or nightly). This showed that after NS RP, treatment with PFMT and early 100 mg sildenafil regular dose (i.e., once daily or nightly) are the best penile rehabilitation strategies with which to improve EF recovery rates at the end of the washout period and that the on-demand dose of PDE5-Is should not be recommended for use in penile rehabilitation [69,70].

The use of tadalafil 5 mg has shown a significant increase in IIEF scores, indicating that its use may aid in the recovery of EF following nerve-sparing RP [70].

A recent prospective study demonstrated that patients undergoing PDE5-I earlier after NS-RALP experienced better EF recovery compared to late-start therapy groups. In this study, a total of 158 patients were treated with PDE5-I after NS-RALP over 2 years. Therapy was started immediately (day 1–2) post-op, early (day 3–14) post-op, or late (after day 14) post-op. The return to baseline EF was 43% for immediate, 36% for early, and 25% for late starts to therapy [33].

Montorsi et al. compared the efficacy of tadalafil 5 mg taken once daily and tadalafil 20 mg on demand versus placebo over a 9-months period in improving unassisted erectile function following NS RP. In this multicenter randomized, double-blind, controlled study of 442 men with normal preoperative EF, the results were as follows: tadalafil once daily = 139, on demand = 142, and placebo = 141. The mean age was 57.9 years of age. At the end of the study (month 9), the proportion of patients with good erection (IIEF > 22) was significantly higher in group which took the tadalafil once daily (25.2%) than in the placebo group (14.2%), while the comparison between tadalafil on demand (19.7%) and placebo was not statistically significant. After a 6-week drug-free washout period, none of the comparisons versus placebo were found to be statistically significant. The same study also identified a significant reduction in penile length loss in the tadalafil once-daily group compared with the placebo group [76].

Previously, the same author conducted a randomized, double-blind, placebo controlled multicenter study, examining the efficacy of tadalafil 20 mg taken on demand in men with ED following bilateral NSRP. In 237 patients (161 tadalafil and 76 placebo) with preoperative normal EF who had undergone NSRP 12 to 48 months before the study, reported a significant important in the tadalafil group in relation to the IIEF and sexual encounter profile questions 2 (successful penetration) and 3 (successful intercourse) compared to placebo [77].

Philippou et al. implemented a systematic Cochrane review of the effect of PDE5-I and intraurethral prostaglandin as monotherapies compared with placebo in post-prostatectomy patients. The review included a total of 1699 post-RP (mostly bilateral NS) patients who were potent pre-op. All interventions started within one month after RP and lasted for a duration between 8 weeks and 12 months, with a washout period between 4–8 weeks. The researchers concluded that there was no benefit of using scheduled PDE5-I over placebo/no treatment or on-demand use in restoring unassisted erectile function [26].

The Fourth International Consultation for Sexual Medicine (ICSM 2015) declares that penile rehabilitation with PDE5-Is is better than placebo; however, there are conflicting data and existing evidence fails to demonstrate improvement in the recovery of spontaneous erections [32].

The only contraindication to the use of PDE5-I is the concomitant use of nitrate-containing medications, which may cause hypotension. The most common side effects of this treatment are transient headaches, flushing, dizziness, dyspepsia, and nasal congestion. However, only 5% of patients discontinued the treatment because of these side effects [2].

The literature on ED treatments after cystectomy is relatively scarce and most data or recommendations have been derived from post-prostatectomy patients.

Moussa et al., in a randomized, double-blinded, prospective study, with 160 potent males with bladder cancer, evaluated the effect on EF (using IIEF score) of early (1 month after NS and NNS RC) pharmacologic therapy using intracorporeal injection (ICI), PDE5I (Sildenafil 50 mg) alone and PDE5-I + ICI compared with a no-treatment group at 12 months of follow-up. After 1 month of surgery, NS and NNS, groups presented severe ED. At 12 months, the NNS group which had been treated with ICI alone and ICI + PDE5-I improved to display moderate and to mild ED, respectively. The NS group remained in the mild ED category with or without any treatment, which was potentially due to neuropraxia recovery. In both groups, NS and NNS, the effect of using PDE5-I alone was not significantly different than that of not using any drug, and the authors concluded that early pharmacotherapy post-NNS RC, using at least ICI, can improve EF [78].

The literature is similarly scarce regarding PDE5-I use in patients after rectal cancer surgery. A recent systematic review, including 253 patients with ED after rectal surgery (82.7% rectal cancer patients and 17.3% surgeries for benign causes), showed that PDE5-I significantly improved IIEF compared to the placebo group at 3 months [79].

### 1.9. Vacuum Erection Devices (VED)

Vacuum devices put negative pressure on the penis, creating a passive blood inflow engorgement of the corpora cavernosa, and use a constrictor ring at the base of the penis to retain the blood within the corpora [80]. This allows for tumescence and enhances penetration capability, as well as cavernosal sinus expansion, smooth muscle and endothelial integrity through anti-hypoxic, anti-apoptotic, and antifibrotic mechanisms, as seen in different studies in rats [9,32] after cavernous nerve injury [9].

Studies have found that VED therapy can significantly improve the peak flow velocity and vascular diameter of the cavernous arteries of patients with organic ED [81].

The effectiveness of on-demand VEDs is very good in men with ED after RP (response rate varies from 60 to 92%) [2,3]. An additional noted benefit is penile length. The shortening of the penis has been described as occurring approximately 4–8 months after RP and can exceed 1–2 cm by 12 months after surgery; daily use of a VED could aid in the preservation of penile length [9,81].

A recent prospective randomized controlled study conducted by Zhung et al., including 91 patients, evaluated EF and penile length following scheduled PDE5-I, VED treatment, and combination therapy after NS RP. Based on their findings, scheduled PDE5-I (5 mg tadalafil once a day) with VED treatment can improve EF at 12 months, whereas VED, alone or combined with tadalafil treatment, can prevent penile length shrinkage after NS RP [81]. This was also found in the meta-analysis of 273 post-RP patients by Feng et al., which showed that the early addition of VED treatment to PDE5-I therapy offers advantages to monotherapy, with PDE5-Ii significantly improving EF and diminishing penile shrinkage [71]. The review of Zippe concluded that after RP, early VED therapy promotes early sexual intercourse, the preservation of penile length, and possibly an earlier return of natural erections [2].

### 1.10. Intracavernosal Injections (ICIs)

ICIs induce relaxation of the trabecular smooth muscle of corpora cavernosa leading to arterial dilation, blood entrapment, and penile erection [9]. The main benefit of ICIs is that they can bypass the damaged nervous pathways and are also effective in patients with vasculogenic ED [2].

ICIs are often used as second-line treatment after the failed use of PDE5-Is. The on-demand use of ICIs (using phentolamine, prostaglandin E1, papaverine, or combinations) is very effective in inducing erections (more than 85%) in post-NS RP ED or non-NS RP patients [9,32]. However, there are also high dropout rates from treatment, ranging from 20–80% [2,82]. These are mainly associated with discomfort or pain [82]. Penile rehabilitation protocols that utilize ICI have shown an improvement in unassisted EF recovery [2,32,83]. A recent systematic review concluded that ICI treatment with use of alprostadil three times per week after RP may be effective in aiding the recovery of spontaneous erections [75].

### 1.11. Intraurethral Therapy with Alprostadil

The medicated urethral system for erections (MUSE) method consists of an alprostadil-containing pellet being inserted into the urethra, absorbed into the corpus spongiosum and subsequently taken up by cavernosal tissue through vascular communications. The overall success rate described is 40% [2]. Raina et al., evaluated the long-term efficacy and compliance of MUSE for ED in 54 patients following RP (NNS and NS) without any other adjuvant therapy. At a follow-up period of 2.3 years, 55% of patients achieved erections sufficient for sexual intercourse (using IEF-15 questionnaire); 48% continued long-term therapy, with a mean use of 2.3 yearsl; and the other 52% discontinued treatment after a mean use of 8.7 months because of insufficient erections (57%), switched to other therapy (14%), a natural return of erections (14%), or urethral pain and burning (14%) [84].

### 1.12. Penile Implants

RP is the most common cancer pelvic surgery associated with ED. Thus the majority of the literature is centered on this group of patients. In males with medically refractory ED, penile prosthesis is the only surgical option [9] and is considered the most effective method with which to achieve on-demand erections, displaying high satisfaction rates and low complications [15,57,58].

Data have shown that despite prosthesis demonstrating efficacy in patients with post-cancer therapy ED, it is generally underutilized. Of a total of 68,500 men receiving treatment for prostate cancer (prostatectomy or external beam radiation), only 0.78% underwent IPP placement [57].

A recent retrospective study identified 31,233 patients from a database of patients treated for prostate cancer (33.1% of patients underwent RP and 66.9% RT). A rate of 44.2% had an ED diagnosis, the diagnoses being more frequent in the prostatectomy group (65.3%) vs. RT group (33.8%) (*p* < 0.001) within 5 years. The median time to ED diagnosis was longer in RT group vs. RP (346 vs. 133 days, *p* < 0.001). Among all patients with ED diagnosis, 2.5% received an IPP. Prostatectomy patients were significantly more likely to receive an IPP vs. the RT group (3.6% vs. 1.4%, *p* < 0.001). ED is not unusual after prostate cancer treatment and this study suggests that IPP implantation is underused [58].

Currently, there are two types of penile prosthesis: malleable and inflatable. Malleable prosthesis generally involves a silicone cylinder with a metal core that allows manipulation of the penis. Inflatable penile prostheses (IPP) are technically more challenging to implant and require a reservoir in which the normal saline is stored while the prosthesis is in the deflated flaccid state [9].

The reservoir of IPP is placed in the space of Retzius (posterior to the transversalis fascia). However, if this has been obliterated due to previous surgeries, it can be placed in ectopic locations, most commonly in submuscular space (anterior to the transversalis fascia and posterior to the rectus abdominal muscle), through a separated incision or the inguinal ring. Alternative locations include the subcutaneous and lateral retroperitoneal space through an incision above the anterior superior iliac spine [15]. The reason for using an ectopic location is to minimize complications such as inguinal hernia, erosion into adjacent viscera, vascular injury, auto-inflation, and infection [9,15,57]. One other option is the use of self-contained 2-piece prosthesis which does not have an independent reservoir.

Satisfaction after a penile prosthesis is very high (over 90%) compared to those receiving pharmacologic treatment, and it is associated with the improvement in EF measured with IIEF-5 [9,15,57,58]. Similarly high satisfaction rates are described in IPP after pelvic radiation without an increase in infections, erosion, or mechanical failures [57,85].

Different authors have described that penile prosthesis has the highest satisfaction and success rates in achieving and maintaining a functional erection compared to other ED treatments [15,58].

Some studies have shown advantages in early IPP implantation, or even in simultaneous IPP implantation at the moment of pelvic cancer surgery. For example, this occurs in patients with a history of ED before pelvic cancer surgery or those who underwent not nerve-sparing surgeries [15]. The proposed advantages of this are the minimization of penile length loss and the immediate, effective treatment of ED [15]. In 1997, Khoudary et al. evaluated the outcomes of IPP simultaneous with NNS ORP in 50 men. Of this cohort, 96% reported the ability to engage in penetrative sexual intercourse after surgery and no infections were found at a follow-up of 1.7 years [86].

In a more recent retrospective study, Mondaini et al. evaluated 10 patients who undergone laparoscopic extraperitoneal RP and simultaneous IPP and after at 2-year follow-up. No difference was found between pre- and post-surgery penile length. They observed one severe complication in a reservoir migration into the bladder after an ulcer formation near a bladder diverticulum. The reservoir was changed through a laparotomy incision [87].

### 1.13. Emerging Therapies

A recent review, conducted by Chung, describes new emerging therapies which can be used to restore erectile function after prostate cancer treatments. The objectives of these therapies are to promote endothelial revascularization and neural regeneration [88]. These therapies include low-intensity extracorporeal shockwave therapy, stem cell therapy, platelet-rich plasma, gene therapy, hyperbaric oxygen, and nerve grafting [11,75]. While conceptually promising, the existing data on ED patients after pelvic cancer surgeries is still too limited to make clear recommendations in this area and further studies are necessary.

## 2. Conclusions

Erectile dysfunction is a common complication after curative treatments for pelvic cancer and it has a significant negative impact on the quality of life of patients. The advent of new, more anatomical surgical techniques that minimize trauma to adjacent tissues, as well as the early start of penile rehabilitation, reflect efforts to minimize ED rates. However, further studies are required to assess the true clinical efficacy of thesemethods.

It is important to inform patients about the real potential risk of ED after pelvic cancer treatment, as well as the existing options to manage it. These include early penile rehabilitation through the progressive use of oral PDE5-I, intracavernous injection therapy, vacuum erection devices, and penile prosthesis as a last resort. The literature on emerging therapies to promote nerve regeneration is limited and research is needed to provide further recommendations.

## Data Availability

Not applicable.

## References

[1] National Institutes of Health (1993). NIH Consensus Conference. Impotence. NIH Consensus Development Panel on Impotence. JAMA.

[2] Zippe C., Nandipati K., Agarwal A., Raina R. (2005). Sexual dysfunction after pelvic surgery. Int. J. Impot. Res..

[3] Chiles K.A., Mulhall J.P. (2016). Management of Erectile Dysfunction after Pelvic Surgery.

[4] Pantelides N.M., Agrawal S., Poullis C., Chetwood A., Winkler M.H. (2011). Erectile dysfunction following radical prostatectomy: A review. Br. J. Med. Surg. Urol..

[5] Jiang N., Wu C., Zhou X., Zhai G., Wu J. (2021). Cavernous Nerve Injury Resulted Erectile Dysfunction and Regeneration. J. Immunol. Res..

[6] Pederzoli F., Campbell J.D., Matsui H., Sopko N.A., Bivalacqua T.J. (2018). Surgical Factors Associated With Male and Female Sexual Dysfunction After Radical Cystectomy: What Do We Know and How Can We Improve Outcomes?. Sex. Med. Rev..

[7] Dean R.C., Lue T.F. (2005). Physiology of Penile Erection and Pathophysiology of Erectile Dysfunction. Urol. Clin. North Am..

[8] Kim S.C., Song C., Kim W., Kang T., Park J., Jeong I.G., Lee S., Cho Y.M., Ahn H. (2011). Factors Determining Functional Outcomes After Radical Prostatectomy: Robot-Assisted Versus Retropubic. Eur. Urol..

[9] Lima T.F.N., Bitran J., Frech F.S., Ramasamy R. (2020). Prevalence of post-prostatectomy erectile dysfunction and a review of the recommended therapeutic modalities. Int. J. Impot. Res..

[10] Podlasek C.A., Meroz C.L., Tang Y., McKenna K.E., McVary K.T. (2007). Regulation of Cavernous Nerve Injury-Induced Apoptosis by Sonic Hedgehog1. Biol. Reprod..

[11] Schoentgen N., Califano G., Manfredi C., Romero-Otero J., Chun F.K.H., Ouzaid I., Hermieu J.-F., Xylinas E., Verze P. (2021). Is it Worth Starting Sexual Rehabilitation Before Radical Prostatectomy? Results From a Systematic Review of the Literature. Front. Surg..

[12] Rogers C.G., Trock B.P., Walsh P.C. (2004). Preservation of accessory pudendal arteries during radical retropubic prostatectomy: Surgical technique and results. Urology.

[13] Box G.N., Kaplan A.G., Rodriguez E., Skarecky D.W., Osann K.E., Finley D.S., Ahlering T.E. (2010). Sacrifice of Accessory Pudendal Arteries in Normally Potent Men during Robot-Assisted Radical Prostatectomy Does Not Impact Potency. J. Sex. Med..

[14] Williams S.B., Morales B.E., Huynh L.M., Osann K., Skarecky D.W., Ahlering T.E. (2017). Analysis of Accessory Pudendal Artery Transection on Erections During Robot-Assisted Radical Prostatectomy. J. Endourol..

[15] Madiraju S.K., Hakky T.S., Perito P.E., Wallen J.J. (2018). Placement of Inflatable Penile Implants in Patients with Prior Radical Pelvic Surgery: A Literature Review. Sex. Med. Rev..

[16] Rawla P. (2019). Epidemiology of Prostate Cancer. World J. Oncol..

[17] Wu S.-Y., Chang C.-L., Chen C.-I., Huang C.-C. (2021). Comparison of Acute and Chronic Surgical Complications Following Robot-Assisted, Laparoscopic, and Traditional Open Radical Prostatectomy Among Men in Taiwan. JAMA Netw. Open.

[18] Kyriazis I., Spinos T., Tsaturyan A., Kallidonis P., Stolzenburg J.U., Liatsikos E. (2022). Different Nerve-Sparing Techniques during Radical Prostatectomy and Their Impact on Functional Outcomes. Cancers.

[19] Xie X., Zhang Y., Ge C., Liang P. (2022). Effect of Brachytherapy vs. External Beam Radiotherapy on Sexual Function in Patients With Clinically Localized Prostate Cancer: A Meta-Analysis. Front. Cell Dev. Biol..

[20] Lee J.K., Assel M., Thong A.E., Sjoberg D.D., Mulhall J.P., Sandhu J., Vickers A.J., Ehdaie B. (2015). Unexpected Long-term Improvements in Urinary and Erectile Function in a Large Cohort of Men with Self-reported Outcomes Following Radical Prostatectomy. Eur. Urol..

[21] Terrier J.E., Masterson M., Mulhall J.P., Nelson C.J. (2018). Decrease in Intercourse Satisfaction in Men Who Recover Erections After Radical Prostatectomy. J. Sex. Med..

[22] Walsh P.C. (1998). Anatomic radical prostatectomy: Evolution of the surgical technique. J. Urol..

[23] Walsh P.C., Lepor H., Eggleston J.C. (1983). Radical prostatectomy with preservation of sexual function: Anatomical and pathological considerations. Prostate.

[24] Kundu S.D., Roehl K.A., Eggener S.E., Antenor J.A.V., Han M., Catalona W.J. (2004). Potency, Continence and Complications in 3,477 consecutive radical retropubic prostatectomies. J. Urol..

[25] Capogrosso P., Vertosick E.A., Benfante N.E., Eastham J.A., Scardino P.J., Vickers A.J., Mulhall J.P. (2018). Are We Improving Erectile Function Recovery After Radical Prostatectomy? Analysis of Patients Treated over the Last Decade. Eur. Urol..

[26] Philippou Y.A., Steggall M.J., Treacy C.L., Hirani S., O’Driscoll S.T., Bakker C.J., Dahm P. (2016). Penile rehabilitation for post-prostatectomy erectile dysfunction. Cochrane Database Syst. Rev..

[27] Lantz A., Bock D., Akre O., Angenete E., Bjartell A., Carlsson S., Modig K.K., Nyberg M., Kollberg K.S., Steineck G. (2021). Functional and Oncological Outcomes After Open Versus Robot-assisted Laparoscopic Radical Prostatectomy for Localised Prostate Cancer: 8-Year Follow-up. Eur. Urol..

[28] Coughlin G.D., Yaxley J.W., Chambers S.K., Occhipinti S., Samaratunga H., Zajdlewicz L., Teloken P., Dunglison N., Williams S., Lavin M. (2018). Robot-assisted laparoscopic prostatectomy versus open radical retropubic prostatectomy: 24-month outcomes from a randomised controlled study. Lancet Oncol..

[29] Ilic D., Evans S., Allan C.A., Jung J.H., Murphy D., Frydenberg M. (2017). Laparoscopic and robot-assisted vs open radical prostatectomy for the treatment of localized prostate cancer: A Cochrane systematic review. BJU Int..

[30] Cao L., Yang Z., Qi L., Chen M. (2019). Robot-assisted and laparoscopic vs open radical prostatectomy in clinically localized prostate cancer: Perioperative, functional, and oncological outcomes. Medicine.

[31] Haese A., Knipper S., Isbarn H., Heinzer H., Tilki D., Salomon G., Michl U., Steuber T., Budäus L., Maurer T. (2019). A comparative study of robot-assisted and open radical prostatectomy in 10 790 men treated by highly trained surgeons for both procedures. BJU Int..

[32] Salonia A., Adaikan G., Buvat J., Carrier S., El-Meliegy A., Hatzimouratidis K., McCullough A., Morgentaler A., Torres L.O., Khera M. (2017). Sexual Rehabilitation After Treatment for Prostate Cancer—Part 2: Recommendations From the Fourth International Consultation for Sexual Medicine (ICSM 2015). J. Sex. Med..

[33] Nathan A., Shukla S., Sinha A., Sivathasan S., Rashid A., Rassam J., Smart S., Patel K., Shah N., Lamb B.W. (2021). Immediate post-operative PDE5i therapy improves early erectile function outcomes after robot assisted radical prostatectomy (RARP). J. Robot. Surg..

[34] Sooriakumaran P., Pini G., Nyberg T., Derogar M., Carlsson S., Stranne J., Bjartell A., Hugosson J., Steineck G., Wiklund P.N. (2018). Erectile Function and Oncologic Outcomes Following Open Retropubic and Robot-assisted Radical Prostatectomy: Results from the LAParoscopic Prostatectomy Robot Open Trial. Eur. Urol..

[35] Ficarra V., Novara G., Ahlering T.E., Costello A., Eastham J.A., Graefen M., Guazzoni G., Menon M., Mottrie A., Patel V.R. (2012). Systematic Review and Meta-analysis of Studies Reporting Potency Rates After Robot-assisted Radical Prostatectomy. Eur. Urol..

[36] Basiri A., de la Rosette J.J., Tabatabaei S., Woo H.H., Laguna M.P., Shemshaki H. (2018). Comparison of retropubic, laparoscopic and robotic radical prostatectomy: Who is the winner?. World J. Urol..

[37] Haglind E., Carlsson S., Stranne J., Wallerstedt A., Wilderäng U., Thorsteinsdottir T., Lagerkvist M., Damber J.-E., Bjartell A., Hugosson J. (2015). Urinary Incontinence and Erectile Dysfunction After Robotic Versus Open Radical Prostatectomy: A Prospective, Controlled, Nonrandomised Trial. Eur. Urol..

[38] Gershman B., Psutka S.P., McGovern F.J., Dahl D.M., Tabatabaei S., Gettman M.T., Frank I., Carlson R.E., Rangel L.J., Barry M.J. (2016). Patient-reported Functional Outcomes Following Open, Laparoscopic, and Robotic Assisted Radical Prostatectomy Performed by High-volume Surgeons at High-volume Hospitals. Eur. Urol. Focus.

[39] Salonia A., Adaikan G., Buvat J., Carrier S., El-Meliegy A., Hatzimouratidis K., McCullough A., Morgentaler A., Torres L.O., Khera M. (2017). Sexual Rehabilitation After Treatment for Prostate Cancer—Part 1: Recommendations From the Fourth International Consultation for Sexual Medicine (ICSM 2015). J. Sex. Med..

[40] Lewis R.W., Fugl-Meyer K.S., Corona G., Hayes R.D., Laumann E.O., Moreira E.D., Rellini A.H., Segraves T. (2010). ORIGINAL ARTICLES: Definitions/Epidemiology/Risk Factors for Sexual Dysfunction. J. Sex. Med..

[41] Alemozaffar M., Regan M.M., Cooperberg M.R., Wei J.T., Michalski J.M., Sandler H.M., Hembroff L., Sadetsky N., Saigal C.S., Litwin M.S. (2011). Prediction of Erectile Function Following Treatment for Prostate Cancer. JAMA.

[42] Kilminster S., Müller S., Menon M., Joseph J.V., Ralph D.J., Patel H.R. (2011). Predicting erectile function outcome in men after radical prostatectomy for prostate cancer. BJU Int..

[43] Broeck T.V.D., Oprea-Lager D., Moris L., Kailavasan M., Briers E., Cornford P., De Santis M., Gandaglia G., Sommer S.G., Grummet J.P. (2021). A Systematic Review of the Impact of Surgeon and Hospital Caseload Volume on Oncological and Nononcological Outcomes After Radical Prostatectomy for Nonmetastatic Prostate Cancer. Eur. Urol..

[44] Cozzi G., Musi G., Monturano M., Bagnardi V., Frassoni S., Jereczek-Fossa B.A., Ferro M., Bianchi R., Mistretta F.A., de Cobelli O. (2019). Sexual function recovery after robot-assisted radical prostatectomy: Outcomes from an Italian referral centre and predicting nomogram. Andrologia.

[45] Mulhall J.P., Kattan M.W., Bennett N.E., Stasi J., Nascimento B., Eastham J., Guillonneau B., Scardino P.T. (2019). Development of Nomograms to Predict the Recovery of Erectile Function Following Radical Prostatectomy. J. Sex. Med..

[46] Agochukwu-Mmonu N., Murali A., Wittmann D., Denton B., Dunn R.L., Montie J., Peabody J., Miller D., Singh K. (2022). Development and Validation of Dynamic Multivariate Prediction Models of Sexual Function Recovery in Patients with Prostate Cancer Undergoing Radical Prostatectomy: Results from the MUSIC Statewide Collaborative. Eur. Urol. Open Sci..

[47] Pellegrino F., Sjoberg D.D., Tin A.L., Benfante N.E., Briganti A., Montorsi F. (2023). Relationship Between Age, Comorbidity, and the Prevalence of Erectile Dysfunction. Eur. Urol. Focus.

[48] Schmid F.A., Poyet C., Rizzi G., Gomolka R.S., Donati O.F., Hötker A.M., Eberli D. (2020). Dynamic contrast enhancement in prostate MRI as predictor of erectile function and recovery after radical prostatectomy. Aging Male.

[49] Iqbal U., Durrani M.M., Elsayed A.S., Hussein A.A., Shigemura K., Fujisawa M., Guru K.A. (2021). Functional outcomes after robot-assisted radical cystectomy: A review of literature. Int. J. Urol..

[50] Benamran D., Phé V., Drouin S.J., Perrot O., Grégoris A., Parra J., Vaessen C., Seisen T., Rouprêt M. (2020). Functional outcomes obtained with intracorporeal neobladder after robotic radical cystectomy for cancer: A narrative review. J. Robot. Surg..

[51] Jue J.S., Mikhail D., Feuerstein M.A. (2021). Systematic review of robotic radical cystectomy functional and quality of life outcomes. Can. Urol. Assoc. J..

[52] Catto J.W., Khetrapal P., Ambler G., Williams N.R., Brew-Graves C., Kelly J.D. (2022). Reply to Andreas Skolarikos’s Words of Wisdom re: Effect of Robot-assisted Radical Cystectomy with Intracorporeal Urinary Diversion vs Open Radical Cystectomy on 90-Day Morbidity and Mortality Among Patients with Bladder Cancer: A Randomized Clinical Trial. Eur Urol. In press. Eur. Urol..

[53] Nukala V., Incrocci L., Hunt A.A., Ballas L., Koontz B.F. (2020). Challenges in Reporting the Effect of Radiotherapy on Erectile Function. J. Sex. Med..

[54] Hunt A.A., Choudhury K.R., Nukala V., Nolan M.W., Ahmad A., Ashcraft K.A., Koontz B.F. (2020). Risk of erectile dysfunction after modern radiotherapy for intact prostate cancer. Prostate Cancer Prostatic Dis..

[55] Gaither T.W., Awad M.A., Osterberg E.C., Murphy G.P., Allen I.E., Chang A., Rosen R.C., Breyer B.N. (2017). The Natural History of Erectile Dysfunction After Prostatic Radiotherapy: A Systematic Review and Meta-Analysis. J. Sex. Med..

[56] Donovan J.L., Hamdy F.C., Lane J.A., Mason M., Metcalfe C., Walsh E., Blazeby J.M., Peters T.J., Holding P., Bonnington S. (2016). Patient-Reported Outcomes after Monitoring, Surgery, or Radiotherapy for Prostate Cancer. N. Engl. J. Med..

[57] Dadhich P., Hockenberry M., Kirby E.W., Lipshultz L. (2017). Penile prosthesis in the management of erectile dysfunction following cancer therapy. Transl. Androl. Urol..

[58] Shen C., Jain K., Shah T., Schaefer E., Zhou S., Fried D., Helmer D.A., Sadeghi-Nejad H. (2022). Relationships between erectile dysfunction, prostate cancer treatment type and inflatable penile prosthesis implantation. Investig. Clin. Urol..

[59] Potosky A.L., Davis W.W., Hoffman R., Stanford J.L., Stephenson R.A., Penson D., Harlan L.C. (2004). Five-Year Outcomes After Prostatectomy or Radiotherapy for Prostate Cancer: The Prostate Cancer Outcomes Study. Gynecol. Oncol..

[60] Frankland J., Wheelwright S., Permyakova N.V., Wright D., Collaço N., Calman L., Winter J., Fenlon D., Richardson A., Smith P.W. (2020). Prevalence and predictors of poor sexual well-being over 5 years following treatment for colorectal cancer: Results from the ColoREctal Wellbeing (CREW) prospective longitudinal study. BMJ Open.

[61] Laurberg J.R., Laurberg V.R., Elfeki H., Jensen J.B., Emmertsen K.J. (2020). Male erectile function after treatment for colorectal cancer: A population-based cross-sectional study. Color. Dis..

[62] Den Oudsten B.L., Traa M.J., Thong M.S., Martijn H., De Hingh I.H., Bosscha K., Van De Poll-Franse L.V. (2012). Higher prevalence of sexual dysfunction in colon and rectal cancer survivors compared with the normative population: A population-based study. Eur. J. Cancer.

[63] Nishizawa Y., Ito M., Saito N., Suzuki T., Sugito M., Tanaka T. (2011). Male sexual dysfunction after rectal cancer surgery. Int. J. Color. Dis..

[64] Towe M., Huynh L.M., El-Khatib F., Gonzalez J., Jenkins L.C., Yafi F.A. (2019). A Review of Male and Female Sexual Function Following Colorectal Surgery. Sex. Med. Rev..

[65] Giglia M.D., Stein S.L. (2019). Overlooked Long-Term Complications of Colorectal Surgery. Clin. Colon Rectal Surg..

[66] Celentano V., Cohen R., Warusavitarne J., Faiz O., Chand M. (2017). Sexual dysfunction following rectal cancer surgery. Int. J. Color. Dis..

[67] Saito S., Fujita S., Mizusawa J., Kanemitsu Y., Saito N., Kinugasa Y., Akazai Y., Ota M., Ohue M., Komori K. (2016). Male sexual dysfunction after rectal cancer surgery: Results of a randomized trial comparing mesorectal excision with and without lateral lymph node dissection for patients with lower rectal cancer: Japan Clinical Oncology Group Study JCOG0212. Eur. J. Surg. Oncol. (EJSO).

[68] Feng D., Tang C., Liu S., Yang Y., Han P., Wei W. (2020). Current management strategy of treating patients with erectile dysfunction after radical prostatectomy: A systematic review and meta-analysis. Int. J. Impot. Res..

[69] Motlagh R.S., Abufaraj M., Yang L., Mori K., Pradere B., Laukhtina E., Mostafaei H., Schuettfort V.M., Quhal F., Montorsi F. (2021). Penile Rehabilitation Strategy after Nerve Sparing Radical Prostatectomy: A Systematic Review and Network Meta-Analysis of Randomized Trials. J. Urol..

[70] Noh T.I., Shim J.S., Kang S.G., Cheon J., Lee J.G., Kang S.H. (2022). Efficacy of Tadalafil in Penile Rehabilitation Started Before Nerve-Sparing Robot-Assisted Radical Prostatectomy: A Double-Blind Pilot Study. Sex. Med..

[71] Qin F., Wang S., Li J., Wu C., Yuan J. (2018). The Early Use of Vacuum Therapy for Penile Rehabilitation After Radical Prostatectomy: Systematic Review and Meta-Analysis. Am. J. Men’s Health.

[72] Madeira C.R., Tonin F.S., Fachi M.M., Borba H.H., Ferreira V.L., Leonart L.P., Bonetti A.F., Moritz R.P., Trindade A.C.L.B., Gonçalves A.G. (2020). Efficacy and safety of oral phosphodiesterase 5 inhibitors for erectile dysfunction: A network meta-analysis and multicriteria decision analysis. World J. Urol..

[73] Pyrgidis N., Mykoniatis I., Haidich A.-B., Tirta M., Talimtzi P., Kalyvianakis D., Ouranidis A., Hatzichristou D. (2021). Effect of phosphodiesterase-type 5 inhibitors on erectile function: An overview of systematic reviews and meta-analyses. BMJ Open.

[74] Goh H.J., Sung J.M., Lee K.H., Jo J.K., Kim K.N. (2021). Efficacy of phosphodiesterase type 5 inhibitors in patients with erectile dysfunction after nerve-sparing radical prostatectomy: A systematic review and meta-analysis. Transl. Androl. Urol..

[75] Nicolai M., Urkmez A., Sarikaya S., Fode M., Falcone M., Albersen M., Gul M., Hatzichristodoulou G., Capogrosso P., Russo G.I. (2021). Penile Rehabilitation and Treatment Options for Erectile Dysfunction Following Radical Prostatectomy and Radiotherapy: A Systematic Review. Front. Surg..

[76] Montorsi F., Brock G., Stolzenburg J.-U., Mulhall J., Moncada I., Patel H.R., Chevallier D., Krajka K., Henneges C., Dickson R. (2013). Effects of Tadalafil Treatment on Erectile Function Recovery Following Bilateral Nerve-sparing Radical Prostatectomy: A Randomised Placebo-controlled Study (REACTT). Eur. Urol..

[77] Montorsi F., Nathan H.P., McCullough A., Brock G.B., Broderick G., Ahuja S., Whitaker S., Hoover A., Novack D., Murphy A. (2004). Tadalafil in the treatment of erectile dysfunction following bilateral nerve sparing radical retropubic prostatectomy: A randomized, double-blind, placebo controlled trial. J. Urol..

[78] Moussa M., Papatsoris A., Chakra M.A., Dellis A. (2021). Erectile dysfunction post radical cystectomy. The role of early rehabilitation with pharmacotherapy in nerve sparing and non-nerve sparing group: A randomized, clinical trial. Arch. Ital. Urol. Androl..

[79] Notarnicola M., Celentano V., Gavriilidis P., Abdi B., Beghdadi N., Sommacale D., Brunetti F., Coccolini F., De’Angelis N. (2020). PDE-5i Management of Erectile Dysfunction After Rectal Surgery: A Systematic Review Focusing on Treatment Efficacy. Am. J. Men’s Health.

[80] Whalen M., Genadiev T. (2019). Preventing erectile dysfunction after radical prostatectomy: Nerve-sparing techniques, penile rehabilitation, and novel regenerative therapies. Prostatectomy.

[81] Liu W., Lu M.-J., Zhang M., Che J.-Z., Liu Y.-D., Wang H.-X., Huang Y.-P., Lv X.-G. (2022). A prospective randomized controlled study on scheduled PDE5i and vacuum erectile devices in the treatment of erectile dysfunction after nerve sparing prostatectomy. Asian J. Androl..

[82] Prabhu V., Alukal J.P., Laze J., Makarov D.V., Lepor H. (2013). Long-Term Satisfaction and Predictors of Use of Intracorporeal Injections for Post-Prostatectomy Erectile Dysfunction. J. Urol..

[83] Mulhall J., Land S., Parker M., Waters W.B., Flanigan R.C. (2005). The Use of an Erectogenic Pharmacotherapy Regimen Following Radical Prostatectomy Improves Recovery of Spontaneous Erectile Function. J. Sex. Med..

[84] Raina R., Agarwal A., Ausmundson S., Mansour D., Zippe C.D. (2004). Long-term efficacy and compliance of MUSE for erectile dysfunction following radical prostatectomy: SHIM (IIEF-5) analysis. Int. J. Impot. Res..

[85] Loh-Doyle J., Patil M.B., Nakhoda Z., Nassiri N., Yip W., Wayne K., Doumanian L., Boyd S.D. (2018). Three-Piece Inflatable Penile Prosthesis Placement Following Pelvic Radiation: Technical Considerations and Contemporary Outcomes. J. Sex. Med..

[86] Khoudary K.P., DeWolf W.C., Bruning C.O., Morgentaler A. (1997). Immediate sexual rehabilitation by simultaneous placement of penile prosthesis in patients undergoing radical prostatectomy: Initial results in 50 patients. Urology.

[87] Mondaini N., Cai T., Sarti E., Polloni G., Gavazzi A., Conti D. (2018). A Case Series of Patients Who Underwent Laparoscopic Extraperitoneal Radical Prostatectomy with the Simultaneous Implant of a Penile Prosthesis: Focus on Penile Length Preservation. World J. Mens. Health.

[88] Chung E. (2021). Regenerative technology to restore and preserve erectile function in men following prostate cancer treatment: Evidence for penile rehabilitation in the context of prostate cancer survivorship. Ther. Adv. Urol..

